# Epigenetics modifiers: potential hub for understanding and treating neurodevelopmental disorders from hypoxic injury

**DOI:** 10.1186/s11689-020-09344-z

**Published:** 2020-12-16

**Authors:** Ana G. Cristancho, Eric D. Marsh

**Affiliations:** 1 Departments of Neurology and Pediatrics, Perelman School of Medicine at the University of Pennsylvania, Philadelphia, USA; 2 Division of Child Neurology, Children’s Hospital of Philadelphia, Philadelphia, USA

**Keywords:** Hypoxia, Brain development, Neurodevelopmental disorders, Epigenetics, DNA methylation, Histone modification

## Abstract

**Abstract:**

**Background:**

The fetal brain is adapted to the hypoxic conditions present during normal in utero development. Relatively more hypoxic states, either chronic or acute, are pathologic and can lead to significant long-term neurodevelopmental sequelae. In utero hypoxic injury is associated with neonatal mortality and millions of lives lived with varying degrees of disability.

**Main body:**

Genetic studies of children with neurodevelopmental disease indicate that epigenetic modifiers regulating DNA methylation and histone remodeling are critical for normal brain development. Epigenetic modifiers are also regulated by environmental stimuli, such as hypoxia. Indeed, epigenetic modifiers that are mutated in children with genetic neurodevelopmental diseases are regulated by hypoxia in a number of preclinical models and may be part of the mechanism for the long-term neurodevelopmental sequelae seem in children with hypoxic brain injury. Thus, a comprehensive understanding the role of DNA methylation and histone modifications in hypoxic injury is critical for developing novel strategies to treat children with hypoxic injury.

**Conclusions:**

This review focuses on our current understanding of the intersection between epigenetics, brain development, and hypoxia. Opportunities for the use of epigenetics as biomarkers of neurodevelopmental disease after hypoxic injury and potential clinical epigenetics targets to improve outcomes after injury are also discussed. While there have been many published studies on the epigenetics of hypoxia, more are needed in the developing brain in order to determine which epigenetic pathways may be most important for mitigating the long-term consequences of hypoxic brain injury.

## Background

The in utero environment is a hypoxic environment compared to ambient conditions. Early in the first trimester, partial pressure of oxygen is as low as 20 mmHg in the placenta and only rises to about 50 mmHg during the second and third trimester (arterial partial pressure of oxygen is 100 mmHg postnatally) [1]. These baseline hypoxic conditions are likely required for normal brain development, as exposure to the ambient environment (i.e., relative hyperoxia) can cause brain injury in premature infants (reviewed in [2]). While exposing the premature brain to high oxygen tension is damaging, further decreasing oxygen levels in pregnancy also leads to significant injury to the developing brain. Worldwide, hypoxic brain injury in preterm and term neonates accounts for many newborn deaths and millions of years lived with disability [3–5]. Neurodevelopmental disorders (NDD) that can be caused by prenatal and perinatal hypoxic injury include developmental and intellectual disabilities, cerebral palsy, autism, and epilepsy.

Hypoxic injury occurs across a spectrum but can be divided into two major categories: chronic and acute. Despite their differing mechanisms of injury, the NDDs caused by chronic and acute hypoxia encompass the entire spectrum of NDDs but are quite variable at the level of the individual patient. Chronic in utero hypoxia is secondary to environmental factors, including high altitude, maternal factors contributing to placental insufficiency (e.g., obesity, smoking, diabetes, or drug use), and fetal factors (e.g. congenital heart disease) [6]. Chronic in utero hypoxia likely contributes to intrauterine growth restriction and is related to increased risk of prematurity; the leading cause of neonatal morbidity and mortality worldwide [4, 5, 7, 8]. There are a number of preclinical models of chronic hypoxia, including rearing pregnant and postnatal animals in hypoxic conditions, exposure to factors that promote placental insufficiency, or mid to late gestation uterine artery ligation [9–11]. These models have demonstrated that chronic hypoxia is correlated to white matter injury similar to what is seen in humans with preterm brain injury.

Hypoxic ischemic encephalopathy (HIE), also known as perinatal asphyxia and neonatal encephalopathy, occurs in 1–6 per 1000 births and is considered to be due to a relatively brief loss of oxygen and nutrients at the end of gestation [3]. The etiology of HIE is varied; it can include sudden events like placental abruption or more indolent events like intermittent umbilical cord compression [3]. It accounts for 23% of neonatal deaths worldwide and nearly half of the surviving children will have abnormal neurodevelopmental outcomes from this injury [5, 12]. The most commonly used model of neonatal HIE is the Rice-Vannucci model where unilateral carotid ligation is performed at postnatal day 8–10 (P8-10) rodents and animals are subsequently exposed to 8–10% fraction of inspired oxygen for 1–2 h (FiO_2_) [9, 13]. This is a hybrid model of HIE and focal stroke but has provided important insights into the pathophysiology of HIE. Other post-natal hypoxia only models have also been used to study neonatal seizures and cerebral palsy-like motor dysfunction [9, 14].

Initial mechanisms of injury for chronic and acute hypoxia are thought to be a result of metabolic dysregulation that leads to significant cell death. In humans, this can only be studied in the most severe cases via post-mortem pathology studies [15], in which the cell death could have occurred after death or is reflective of an atypically severe process. If there is significant cell death, estimated indirectly in surviving children by the burden of diffusion restriction seen in brain magnetic resonance imaging, there is likely to be significant neurologic sequelae from injury [15, 16]. However, it is clear even if there is not significant cell death, surviving neurons and glial cells continue to have abnormal structure and dysfunction long after in utero hypoxic brain injury, which dictates long-term neurologic outcomes [17, 18]. Long-lasting injury to surviving cells is likely due to the unique combination of injury superimposed on a critical period of brain development.

An attractive unifying hypothesis linking prenatal hypoxia to the persistent functional and structural deficits of many different cells in the brain is that prenatal hypoxia permanently alters the epigenome [19–22]. The epigenome is the profile of transcription factors and histone and DNA modifications that dictate cell identity and function without altering the genetic code and it is extensively regulated during normal development [19–22]. Several mutations in epigenetic modifiers can lead to a variety of NDDs [23, 24], and the epigenome can be regulated extensively by environmental inputs, such as perinatal stressors [19–22]. This review will give a brief overview on epigenetic mechanisms important for brain development, with focus on the potential roles of epigenetic modifiers in dictating outcomes after hypoxic brain injury during development that overlap with genetic NDDs, and opportunities to use epigenetics to predict neurodevelopmental outcomes or as therapeutic targets for improving neurodevelopmental outcomes.

### Primer on epigenetic modifications

**Fig. 1:**
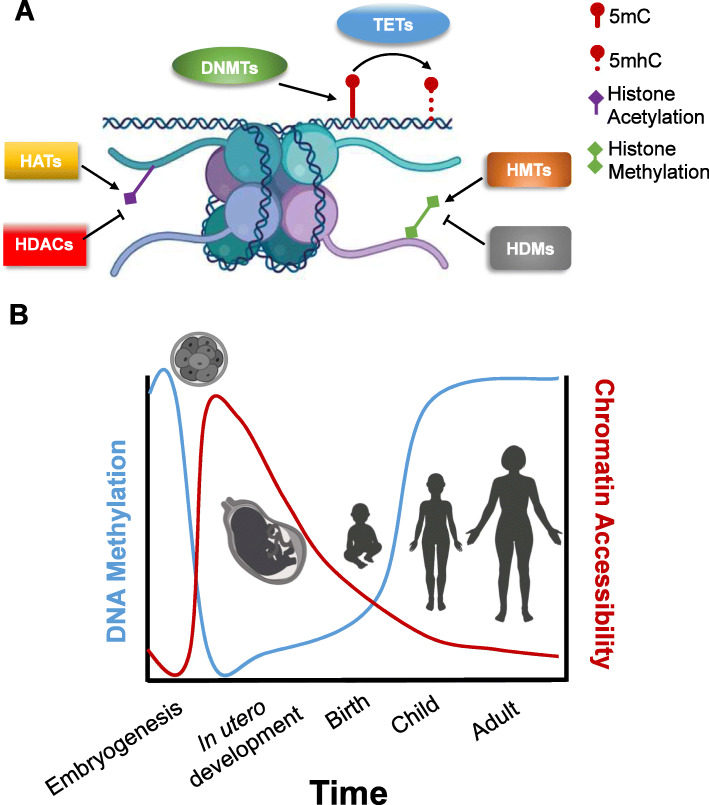
There are multiple layers of epigenetic gene regulation that are tightly regulated during development. **a** The epigenetic landscape is dictated by a number of modifications to DNA and histones as indicated in legend. The primary modification to DNA is DNA methylation on the nucleotide cytosine (5′ methylcyrtosine, 5mC) by DNA methyltransferases (DMNTs). Methylated DNA can also be demethylated in a multistep process. The first step in this process is conversion of 5mC to 5′-hydroxymethylcystosine by ten-eleven translocation family proteins (TETs). At the histone level, histones can be covalently modified at multiple locations. The most common modifications are acetylation and methylation (lesser understood and not shown are histone phosphorylation and ubiquitination.) Histone acetylation is regulated by histone acetyltransferases (HATs) and histone deacetylases (HDACs). Histone methylation is regulated by histone methyltransferases (HMTs) and histone demethylases (HDMs). The balance of histone methylation of demethylation at different histone residues dictates chromatin accessibility. **b** DNA methylation and chromatin accessibility are tightly regulated during the course of brain development throughout the lifespan. While there are not direct studies comparing DNA methylation and chromatin accessibility in the same brain samples during development, here we schematize a summary of known studies that demonstrate a near inverse relationship between extent of DNA methylation (blue line) and chromatin accessibility (red line) over time

The epigenome plays a critical role in development by orchestrating which genes are active during all stages of maturation [25, 26]. DNA methylation and histone modifications, two of the predominant epigenetic modifiers (often called epigenetic marks), dynamically change starting from embryogenesis [27, 28] (Fig. 1a). Coordination of these epigenetic marks throughout the course of development forms identifiable epigenetic trajectories that are thought to be critical for cell maturation and specification [25, 29, 30] (Fig. 1b).

#### DNA methylation

DNA methylation is an extensively regulated epigenetic mark during development and throughout the life of a cell [31–33]. DNA methylation occurs at the 5′ position of the nucleotide cytosine (5′-methylcystine, 5mC). DNA methyltransferases (DNMTs) are required for methylation. Early research hypothesized that this mark was irreversible (reviewed in [34]). More recently, though, demethylation has been demonstrated to be catalyzed by ten-eleven translocation family proteins (TETs); this enzyme promotes conversion from 5mc to 5′-hydroxymethylcytosine (5hmC), which can ultimately actively be converted to cytosine through thymine DNA glycosylase-mediated base excision repair (reviewed in [35–37]). Demethylation by TETs are vital immediately after fertilization when both the genome of the maternal and paternal DNA are almost completely demethylated to become totipotent stem cells [38, 39]. TETs may play an important role in mature brain function as well; analysis of human postnatal tissues reveals that the brain has the highest levels of 5hmC content pointing to a lifelong dynamic regulation of methylation [40]. Supporting these ideas are studies by Mills and colleagues that demonstrate that 5mC and 5hmC are dynamically changing during human fetal brain development both globally and at specific loci [41, 42].

The exact role of DNA methylation in epigenetic regulation of transcription has become more complicated in recent years. The most extensively studied form of methylation occurs when cytosine is directly followed by guanine (known as CpG or CG sites) (reviewed in [28, 31]). When CG sites are clustered, they are known as CG islands. Methylation of CG islands is typically associated with gene silencing [28, 31]. However, in the brain there is also extensive non-CG methylation, particularly in neurons (known as CH where the “H” nucleotide = A/C/T) [32, 43]. While in some locations, methylated CH sites are associated with CG islands and DNA repression, in other areas they are associated with genes escaping X-inactivation and transcriptional activation [32, 43] (and reviewed in [44, 45]). During the course of brain development and maturation, there is a gradual accumulation of 5mC at CG and CH sites; thus, the role of DNA methylation in modulating transcription and neurodevelopmental disease is likely related to the balance between 5mC and 5hmC at specific CG and CH sites [32].

#### Histone modifications

Histone modifications form a possibly more complicated regulation of transcription than DNA methylation due to the extensive number of modifications that can take place on the nucleosome. In brief, nucleosomes are comprised of two copies each of four histone subunits: histone 2A (H2A), histone 2B (H2B), histone 3 (H3) and histone 4 (H4). A linker protein, histone 1 (H1), is recruited between them. When DNA is “tightly wound” around these structures, it is not accessible to transcriptional activators. However, covalent modifications to specific lysines (K) or arginines (R) to these histones can alter how tightly DNA is bound to the genome. For example, dimethylated (me2) or trimethylated (me3) histone 3 lysine 9 (H3K9) and H3K27me3 are marks for transcriptional repression but acetylation (ac) of H3K9 and H3K27 are associated with transcriptional activation (reviewed in [21, 25, 46]).

The ultimate read out from these modifications is chromatin accessibility. Greater accessibility is correlated to increased transcriptional activation since DNA is not only more accessible to transcription factors, but also to the transcriptional machinery, such as RNA polymerase [21, 25, 46]. Accessibility can be profiled in the genome through high throughput sequencing techniques which assess level of expression of genes around known epigenetic marks or more globally. Using these techniques in an organoid model of forebrain development, Pasca and colleagues recently demonstrated that chromatin accessibility patterns change throughout development differently in neurons and glia [30]. In particular, during early development of these organoids, there is a burst of increased chromatin accessibility, which may be related to periods of relative demethylation that has been observed in embryonic stem cell systems. As cells continue to mature, cell-type specific motifs continue to remain more accessible. For example in neurons, motifs near regulators of synaptic function remained more open in mature cells than motifs near genes important for neural differentiation or proliferation [30]. Interestingly, this early increase in accessibility was more obvious in neuron lineages than in glial lineages. It is unclear if this discrepancy is related to organoid differentiation of different glial cells or truly reflects in vivo development.

Genome accessibility is regulated by a large number enzymes that add/remove these covalent modifications, including histone acetyltransferases (HATs), deacetylates (HDACs), histone methyltransferases, and demethylases. Each of these proteins regulate the histone code through association with transcriptional activators or repressors (extensively reviewed in [25, 26, 47, 48]). The complex regulation of this intricate histone code allows for exquisite regulation of transcription in response to developmental and environmental stimuli that are important to understand neurodevelopmental outcomes from genetic and acquired disorders.

### Epigenetics in brain development—lessons from neurogenetics

The advent, and clinical implementation, of massively parallel sequencing has resulted in a dramatic evolution in our understanding of the genetics of neurodevelopmental disease. By discovering the genetic etiologies of intellectual disability, autism, and epilepsy, we have obtained important insights into the pathways that are critical for brain development and function.

Interestingly, while over 1000 genes with de novo mutations in patients with NDDs have been discovered, a review by Eichler and colleagues highlighted that there are three pathways that have emerged as central nodes for mutations in patients with NDDs: chromatin remodeling, wingless (WNT) signaling, and synaptic function [49]. Two of these pathways, chromatin remodeling and WNT signaling, are direct modulators of the epigenome and transcriptional regulation thus highlighting the importance of epigenetics in neurodevelopmental disease. Neuronal activity, albeit more indirectly, also regulates the epigenetic landscape in neurons, thus strengthening the hypothesis that regulation of the epigenome is central to normal brain development [50, 51].

Several of the epigenetic modifiers in which mutations (pathogenic variants) are associated with NDDs are also regulated by hypoxia or can regulate the hypoxic response (Table 1). This link between epigenetic modulating genes associated with the NDDs and normal brain development, suggests that epigenetic changes due to environmental insults (in this case hypoxia) may be vital to understanding the mechanism of HIE. Indeed, these epigenetic pathways may be key to improving our treatments of NDDs from multiple etiologies. Below we will highlight lessons from genetic disorders in DNA methylation and histone modifications that may provide insight into the hypoxic response during prenatal and perinatal injury.

**Table 1:** List of epigenetic modifiers that are mutated in children with developmental disorders but have also been described as mediators of the hypoxic response

Overlap between genetic developmental disorders and hypoxic response		
DNA methylation	Histone modifications	
DNMT3A	CHD7	HDAC4
DNMT3B	CHD8	KMT2D
MeCP2	p300	KDM6A

### DNA methylation in genetic NDDs

In addition to their role during embryonic development, there is extensive evidence from genetic disorders that dynamic methylation and demethylation processes continue to have important functions in postnatal brain function. First, DNMT3A, which is a rare cause of a syndromic NDD, is expressed at high levels in post-mitotic neurons and oligodendrocytes throughout maturation into adulthood [52, 53]. *DNMT3A* null mice have decreased survival in the early postnatal period, and mice lacking *DNMT1* and *DNMT3A* in post-mitotic neurons have deficits in hippocampal size, learning, and memory [54]. DNMT3A but not DNMT1 is required for the maintenance of CH methylation in mature cortex [43]. DNMT3B is also mutated in syndromic neurodevelopmental disease, although it seems to have a limited role in murine brain development so we have a more limited understand of its role in brain development and function [55]. Interestingly, there are no TETs mutated in human disease and TET-deficient mice are not embryonic lethal, which may indicate these are critical enzymes specifically for human development or there are other enzymes that can compensate for loss of function (reviewed in [35, 36]).

Recognition of DNA methylation binding is also important for development. Methyl-CpG-binding protein 2 (MeCP2), which is mutated in Rett Syndrome—one of the most common causes of severe intellectual disability with autistic features in girls (but rarely in boys)—has a high affinity for binding at methylated DNA at CG islands and recruiting transcriptional repressors for gene silencing in the brain [56, 57]. However, in a recent review, Kinde et al. described extensive work on how MeCP2 can also bind CH sites and 5hmC. This CH binding suggests that MeCP2 may have a diverse capacity to finely regulate transcription in the post-mitotic neurons [45] further highlighting the importance of different forms of epigenetic marks during development and in the mature brain.

#### Histone modifications in genetic NDDs

Different mutations that have been found in patients with NDDs implicate almost every aspect of histone structure and remodeling as important factors for brain development [49]. One of the first described epigenetic gene mutations in autism was in the chromatin domain helicase DNA-binding proteins (CHD) 7 and 8. CHD7 and CHD8 are ATP-dependent chromatin remodeling proteins with different functions despite their similar names. CHD7, which is associated with CHARGE syndrome (coloboma of the eye, heart defects, atresia of the nasal choanae, retardation of growth and development, genital and urinary abnormalities, and ear abnormalities and deafness), co-localizes to active genes and is important for hippocampal neurogenesis [58, 59]. By contrast, CHD8 is mutated in non-syndromic autism and is thought to recruit H1 and lead to transcriptional repression of targets like p53 and β-catenin during development, implicating histone organization in regulating brain function and directly linking chromatin remodeling to WNT pathway regulation [60, 61].

Furthermore, modifiers of the histone modification code (both acetylation and methylation) have been implicated in children with genetic NDDs. Rubinstein-Taybi syndrome, a craniofacial syndrome with intellectual disability, is associated with the histone acetyltransferase (HAT) p300 [62]. This highly conserved HAT is near ubiquitous and binds to dozens of transcription factors to increase chromatin accessibility and promote gene expression, giving it a critical function in a number of developmental processes including brain development (reviewed in [63, 64]). More recently histone deacetylase 4 (HDAC4) was implicated in children with intellectual disability [65]. Lastly, Kabuki syndrome, a syndromic NDD with constellation of distinct facial and skeletal anomalies, consists of mutations in one of two opposing regulators of histone methylation: lysine-specific methyltransferase 2D KMT2D and the Jumonji C-domain protein lysine-specific demethylase 6A (KDM6A) [66]. The similar phenotype between these functionally opposing factors highlights that the epigenetic landscape needs balanced regulation of epigenetic marks for normal brain development and function.

Given the rapid expansion of this field, as we gain further insight into genetic causes of NDDs and more insights into the role of epigenetic modulators in cell type-specific brain development we will better understand how these epigenetic mechanisms regulate cell-type specific functions in the mature brain, during development, and even how these modifiers effect the brain’s response to hypoxic injury.

### Epigenetics in hypoxic brain injury

In addition to the epigenetic progression that occurs as part of normal development, the epigenetic landscape of the developing brain is responding to external signals from the maternal-placental environment, including maternal diet or stress and placental health (reviewed in [67]). Epigenetic modifiers are also critical in mediating the response to a hypoxic in utero environment. The canonical response to a hypoxic environment is induction of hypoxia inducible factor 1 alpha (HIF1α), a transcriptional activator that is stabilized by hypoxia and critical to a cell’s compensatory response to low oxygen conditions [68]. HIF1α targets are diverse and are responsible for promoting angiogenesis and hematopoiesis, regulating metabolic demand, and increasing nutrient uptake to preserve cell survival [68]. The role of HIF1α in HIE as a protective or deleterious factor has been controversial with reports of conflicting reports [69, 70]. These differences are likely due to experimental issues, such as timing of the insult and when HIF1α is stabilized or inhibited, indicating there may be a delicate balance between HIF1α requirements for normal development and HIF1α dose-dependent effects between compensatory and pathologic responses to hypoxic insult.

Regulation of HIF1α activity by epigenetic modifiers is critical to the response of many cell types to hypoxic insult (extensively reviewed in by multiple sources [68, 71–73]. The HIF1α promoter itself may be regulated by CG and CH methylation, contributing to its abundance in a cell [74]. Additionally, the HIF1α response element (sequence 5′-RCGTG-3′) contains a CG site and methylation at these sites dramatically alters the affinity of HIF1α to these binding sites [75]. HIF1α also directly upregulates expression of several of the Jumonji chromatin demethylases [76]. While methylation of HIF1α targets have not been studied in the developing brain, accessibility to HIF1α binding sites during different stages of development and in different cell types may account for the ability of the brain to compensate for hypoxic injury, particularly in regulating Jumonji C-domain proteins. Many of the same epigenetic modifiers and processes implicated in children with genetic causes of NDDs have also been implicated in the cellular response to hypoxia, including some evidence that they may be involved in hypoxic brain injury.

#### DNA methylation and hypoxic injury

Numerous lines of evidence point to the impact of hypoxia on DNA methylation. The interplay between hypoxia and methylation have largely been studied in the context of chronic hypoxic exposure, such as in high altitude, sleep apnea, or cancer. However, even relatively brief hypoxia exposure in cultured hippocampal neurons leads to lasting changes in DNA methylation [19]. Different regions of the genome are hypermethylated and hypomethylated in the setting of hypoxia. DNMT3B is induced in ovine uterine arteries by chronic gestational hypoxia and associated with a hypermethylation of BKCa channel beta-1 subunit, a potassium channel subunit [77]. It is unknown if the fetal brain has similar changes in response to changes in oxygen tension in the placenta. Furthermore, populations that live at high altitude have regions of increased and decreased methylation at genes associated with compensatory response to hypoxia [78]. Prenatal stress as also been associated with changes in methylation. One of the changes that was observed was hypomethylation of p300 in children who experience prenatal stress, raising the question as to whether prenatal stress dysregulates this intellectual disability gene [79]. It is important to note that human studies are from peripheral blood samples, which may demonstrate a significantly different pattern of methylation than what is observed in brain tissue. In tumor cells, hypoxia is associated with general hypermethylation due to the repression of TET activity. However, chronic in utero hypoxia is related to global DNA hypomethylation of the brain in rodents, indicating the need for further studies in the developing brain to determine the tissue-specific response to hypoxic injury [80].

Abnormalities in DNA methylation may provide a mechanistic explanation for the epidemiological link between chronic and acute hypoxic insult to the developing brain. One potential link is through regulation of MeCP2 activity. The role of hypoxia has also been studied in the setting of Rett syndrome since children with Rett syndrome have irregular breathing patterns possibly leading to transient post-natal hypoxia [81–83]. Children with Rett and MeCP2-deficient mice have increased oxidative stress burden indicating metabolic dysfunction could play a role in pathophysiology of this disorder [81, 84, 85]. Antenatal risk factors in HIE include factors such as maternal illicit drug use and excess weight gain, which would place the fetus in an environment of chronic hypoxia prior to an acute insult [86]. To test the contribution of chronic in utero hypoxia as a primer for worsening HIE outcomes, Zhang and colleagues exposed pregnant rats to continuous 10% FiO_2_ from embryonic day 15–21 and then performed Rice-Vannucci paradigm at P10 [87]. Animals exposed to chronic prenatal hypoxia have significant worsening of infarct size that is related to methylation and subsequent repression of the glucocorticoid receptor by MeCP2 [87]. Consistent with this finding, pretreating rats with azacitidine, a DNA methylation inhibitor, also predisposes rats to larger infarct volumes after HIE [80]. Worsening of an acute hypoxic injury after chronic hypoxia may be unique to the developing brain as hypoxic preconditioning protocols in adult stroke models decreases infarct volume [88].

However, acute hypoxia alone may also regulate MeCP2 activity. In adult transient ischemia there is an increase in MecP2 levels [89]. Hypoxia-induced seizures in neonatal rats leads to increase MeCP2 phosphorylation at serine 421 and treating seizures abrogates MeCP2 phosphorylation [90]. This post-translational modification decreases spine density in the setting of MeCP2 overexpression, a pathological state [91]. Future studies of hypoxia in the setting of MeCP2^S241A^ mutant would help determine if long-term deleterious effects of hypoxia-related neonatal seizures can be mitigated by inhibiting MeCP2 phosphorylation.

These findings bring forth interesting questions. First, are there other regions of the genome that are effected by chronic antenatal stress that “prime” the response to more significant hypoxic insult later in gestation? Second, since the in utero environment is more hypoxic at baseline, does HIE have other direct effects on methylation status of neurons and support cells without priming by a prenatal stressor? Lastly, do chronic and acute hypoxia differentially effect the balance between 5mC and 5mhC at CG or CH sites and what is the final effect skewing of the methylation status have on gene expression in the human brain? Better understanding these questions may allow us to determine if intervening on antenatal factors affect neurodevelopmental outcomes in HIE by minimizing damage from an acute hypoxic insult.

#### Histone modifications and hypoxic injury

By regulating transcription through HIF1α, hypoxia undoubtedly has significant effects on histone mark patterns at the loci of its targets. Many of the histone modifying genes have been shown to have important roles in regulating the hypoxic response in various cell types. CHD7, which regulates neurogenesis, is repressed in hypoxic-ischemic microenvironments of glioblastoma cells, indicating it might regulated in other hypoxic states during development [59, 92]. KMT2D hypomorphic cells and knockout mice have an increased stabilization of HIF1α but decreased expression of hypoxia genes, indicating the KMT2D may be a critical component of the HIF1α transcriptional activation machinery [93]. While there have not been studies of specific HDACs in models of HIE, the pan HDAC inhibitor sodium butyrate in adult stroke models promotes neurogenesis in the hippocampus [94, 95]. Sodium butyrate may be mediating this affect as an anti-inflammatory or by promoting expression of brain-derived neurotrophic factor in neurons and support glial cells [95, 96].

Recently, there has been exciting work on the role of HIF1α-independent regulation of chromatin by hypoxia. Histone methylation is increased by hypoxia in a number of cell lines independent of HIF1α stabilization [97]. This regulation may be mediated by Jumonji C domain proteins, which are in a superclass of proteins known as 2-oxoglutarate and Fe (II)-dependent dioxygenases (2-OGDs) (reviewed in [98]). The 2-OGDs superclass also includes the prolyl hydroxylase required for HIF1α stabilization under hypoxic conditions, thus other enzymes from this class also have the ability to directly respond to hypoxic conditions. KDM6A was recently described as a 2-OGD protein capable of acting as an oxygen sensor independent of HIF1α, making it an attractive candidate to study in the setting of hypoxic brain injury [99].

While there are not many studies of most of these epigenetic modifiers in pre-natal or perinatal hypoxic brain injury, we now have ample evidence justifying studies in hypoxic brain injury. By using clinically relevant disease models, we may (1) acquire a more precise understanding of the roles of epigenetic modifiers in brain development, (2) understand how the modifiers may be disrupted differentially during develop by environmental insults, and (3) develop novel therapeutic strategies.

### Other opportunities for future studies and interventions

**Fig. 2:**
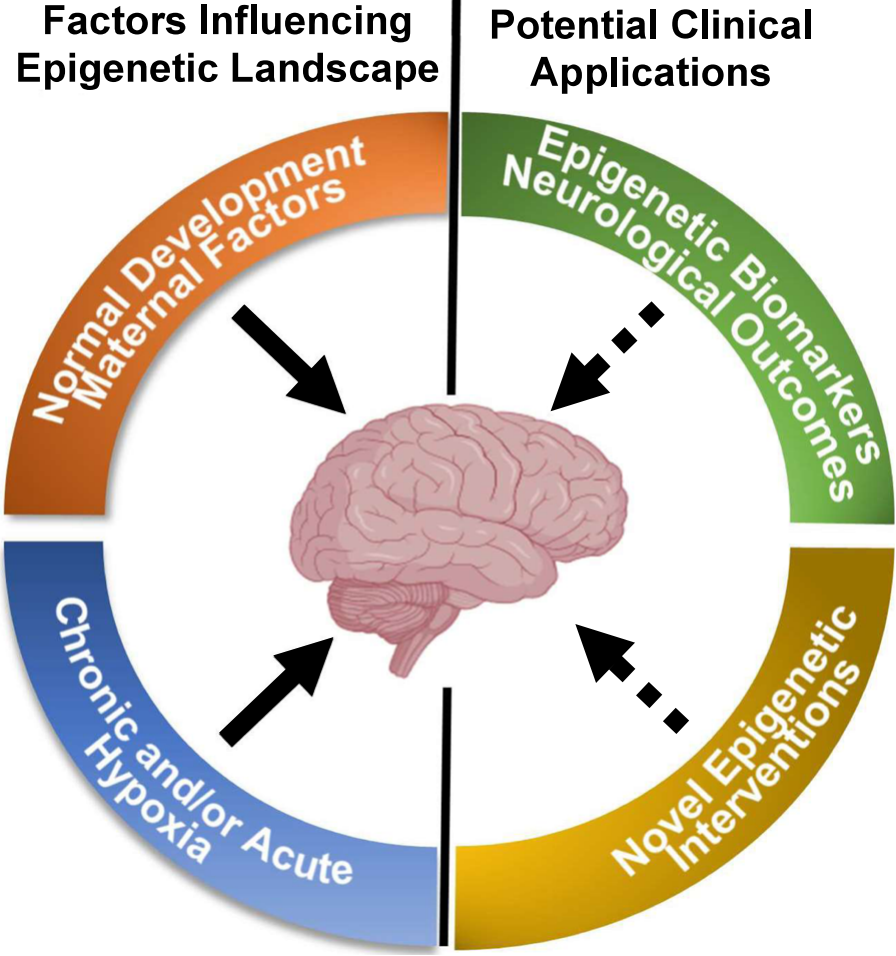
Epigenetic modifiers may allow for clinical insight into developmental hypoxic brain injury. The developing human brain is influences throughout the course of development by a number of extrinsic (i.e., maternal/placental factors) and an intrinsic developmental program. Chronic and acute hypoxic stress during in utero development are also likely to alter the epigenome, and effect the ultimate maturation of function of the brain leading to neurodevelopmental disorders. By further understanding the role of the epigenome in brain development and mature brain function, we hope to determine better biomarkers for neurodevelopmental disease after prenatal injury. We can also take advantage of a number of compounds that have been developed to modulate the epigenome for other diseases to rapidly develop novel therapeutics to improve cognitive outcomes from hypoxic injury

Epigenetic modifiers have important roles in a number of other diseases, including cancer, metabolism, and cardiovascular disease [31, 72, 100, 101]. Therefore, as we increasingly appreciate the important roles of epigenetic regulation in normal brain development and function, we are able to leverage a number of tools that have already been developed by other fields to expedite clinical translation of epigenetics as both a biomarker of disease and therapeutic target (Fig. 2).

#### Epigenetic biomarkers

Methylation abnormalities in the setting of perinatal disease have recently garnered increased interest as a biomarker for neurodevelopmental outcome/disease-severity in the neonatal period as well as in the long-term. Preterm neonates that had atypical performance on the NICU Network Neurobehavioral Scale had differential methylation at almost 30 loci in blood samples [102]. Consistent with the possibility that peripheral DNA methylation status can be a biomarker for neurodevelopmental outcomes, in a small study of newborn blood spots in monozygotic twins that eventually were discovered to be discordant for cerebral palsy, researchers identified differentially methylated regions between affect and unaffected siblings [103].

Furthermore, the methylation clock has been used as a measurement for maturation and can be used to approximate gestational age at birth as well as biology age, known as the “epigenetic age” [104–106]. In humans, epigenetic age is slowed in umbilical cord blood after prenatal stress [79]. Changes from prenatal stress may be sustained at some portions of the genome for up to 13 years [107]. Studies that validate these markers in different populations and across different ethnic groups are still needed. Importantly, it is not clear if these methylation differences can be modified in order to be used as markers for potential therapeutic interventions.

#### Epigenetics as a target in neurodevelopmental disease

Epigenetic modifiers are highly tractable to pharmacologic interventions, with many medications currently under development for cancers and neurodegenerative disorders (reviewed in [23, 100, 108]). Epigenetic targeted therapeutics have successfully rescued neurocognitive deficits in models of Rett and Kabuki syndromes [109, 110]. Interestingly, approach in neither disease has focused on directly modifying the genome. For Rett syndrome, MeCP2 overexpression can lead to as many neurological sequelae as the deletion, therefore most approaches have focused on modulation of diverse downstream pathways, including signaling and metabolic pathways [110]. In a preclinical trial for Kabuki syndrome, Dietz and colleagues treated *Kmt6d*-deficient mice with the HDAC inhibitor AR-42 in the juvenile period, reasoning that the mutation led to a favoring of closed chromatin state that would be counteracted with HDAC inhibition. Remarkably, despite the mutation being present throughout development, AR-42 rescued memory deficits in mutant mice [109]. Similarly, neurogenesis is impaired in in *CHD7*-null mice but can be rescued by increasing the amount the animals exercise on a voluntary running wheel [59]. Exercise is thought to promote epigenetic remodeling of the promoter of brain derived neurotrophic factor, an important regulator of neurogenesis and myelination [111–113]. These studies provide compelling evidence that epigenetic modulators may be viable targets for treating neurocognitive deficits and that the window for treating NDDs may extend past early development.

HDACs in general are attractive targets for hypoxic brain injury. However, many studies are needed to determine the effects of medications like sodium butyrate on neurocognition after administration in the perinatal period. Valproic acid, a commonly used anti-epileptic that is also an HDAC inhibitor, is controversial in the neonatal period [114]. Some studies report a neuroprotective effect in rodents in the setting of HIE, while other report significant increases in cell death and behavioral deficits in control animals when the drug is administered in the neonatal period [115, 116]. It is important to note that all valproic acid studies used 2–5 times the upper limit of valproic acid that is typically used in patients, limiting their interpretation for the clinical setting. However, sodium butyrate has also been shown to induce senescence and apoptosis in cancer cells, thus using this medication in neonates requires significant testing in preclinical models of chronic hypoxia and HIE [117].

TETs may also be an attractive target to study in prenatal brain hypoxic brain injury. In a model of adult stroke, Tet3 was induced in the penumbra of infarcted tissue [118]. *Tet3*-deficiency was associated with worsening of the infarct. Thus, stabilizing these proteins may be a viable strategy for neuroprotection. TET induction would be an attractive hypothesis to explain the global hypomethylation of DNA seen after chronic in utero hypoxia if it indeed is not critical to brain development as suggested in animal studies. However, further studies are needed to determine whether this hypomethylation phenomenon is due to a change in the balance of DNMTs and TETs and if it is indeed a protective mechanism as seen in stroke [80]. As we continue to unravel the mechanisms underlying TETs and other epigenetic modifiers, we will likely be able to develop novel intervention strategies that are targeted appropriately to different ages of brain maturation.

## Conclusions

In summary, the regulation of many aspects of the epigenome are critical for normal in utero brain development and continue to be important in the mature brain. The continually emerging clinical genetic data linking mutations in epigenetic-involved genes in children with various genetic etiologies of the NDDs, including autism, epilepsy, and intellectual disability emphasizes the importance of these epigenetic process in normal brain development. Mechanistic studies have illustrated that these modifiers have multiple roles in brain development as well as in modulating the brain’s response to environmental stressors, such as pathologic chronic and acute in utero hypoxia. By using an array of tools, including genetic models and high throughput profiling, we have an opportunity to understand which epigenetic modifiers are most important for regulating hypoxic injury and which pathways are most affected by insults to the developing brain. Ultimately, the pathways that regulate and are regulated by the epigenome are potential hubs for understanding and modulating neurodevelopmental sequelae from hypoxic brain injury.

Further studies with current technologies will allow us to understand the roles of the epigenome in mediating both injury and recovery from hypoxia in specific cell types. Cell type-specific models and single-cell profiling studies may allow us to understand how the epigenetic modifiers described in this review may change throughout development, how these same regulators may be affected by hypoxia, and how hypoxia leads to neurological disease. Studying the cell-specific effects of candidate epigenetic controller genes, will determine which cell types are most affected by hypoxia and could be targeted for intervention. Gaining a temporal understanding of the role of these candidate epigenetic controller genes, will allow for a tailored understanding of both the pathogenesis and treatment of either chronic or acute hypoxic injury. Systematic study of therapies targeting the epigenome by globally altering the balance of DNA accessibility has the potential to provide a vast clinical pipeline of agents to improve neurological outcomes.

## Data Availability

Not applicable

## Abbreviations

2-OGDs: 2-oxoglutarate and Fe (II)-dependent dioxygenases
5mc: 5′-methylcytosine
ATM: Ataxia telangenctasia mutated protein
CHARGE: Coloboma of the eye, heart defects, atresia of the nasal choanae, retardation of growth and development, genital and urinary abnormalities, and ear abnormalities and deafness
CHD: Chromodomain helicase DNA-binding protein
DNMT: DNA methyltransferases
H1: Histone 1
H2A: Histone 2A
H2B: Histone 2B
H3: Histone 3
H4: Histone 4
HAT: Histone acetyltransferase
HDAC: Histone deacetylase
HIE: Hypoxic ischemic encephalopathy
HIF1a: Hypoxia inducible factor 1 alpha
K: Lysine
KDM6A: Lysine-specific demethylase 6A
KMT2D: Lysine-specific methyltransferase 2D KMT2D
R: Arginine
TET: Ten-eleven translocation family proteins
WNT: Wingless
